# Indirect evidence of an early mating advantage in wild cooperatively breeding male banded mongooses

**DOI:** 10.1038/s41598-024-80518-8

**Published:** 2025-01-09

**Authors:** Graham Birch, Michael A. Cant, Hazel J. Nichols, Magali Meniri, Robert Businge, Francis Mwanguhya, Jonathan D. Blount

**Affiliations:** 1 Centre for Ecology and Conservation, Faculty of Environment, Science & Economy, University of Exeter, Penryn Campus, Cornwall, TR10 9FE UK; 2 Department of Biosciences, Swansea University, Singleton Campus, Swansea, SA2 8PP UK; 3 Banded Mongoose Research Project, Queen Elizabeth National Park, Kasese, Uganda

**Keywords:** Cooperative breeder, Mate competition, Sperm competition, Polyandry, Paternity, Sperm precedence, Copulatory plug, Ecology, Physiology, Zoology, Ecology

## Abstract

Promiscuous females reduce male reproductive control. Males can attempt to monopolise access to these females, but distractions and sneaky rivals mean extra copulations cannot always be blocked. By mating first, males can obtain a headstart in sperm competition, but this may be negated by sperm storage and cryptic female choice mechanisms. We carry out an indirect rare test of an early mating advantage in a population of free-living wild animals. Using Bayesian GLMM analysis of a long-term life history database spanning 17 years, we show that banded mongoose males who interacted with females in earlier days of oestrus had a higher chance of siring their offspring compared with later rivals. An early mating advantage would intensify initial male-male competition and hence selection for male choice, as any initial mistake identifying preferred mating partners could see paternity lost to rivals.

## Introduction

For animals that live in mixed sex societies, group members of the same sex are both allies and reproductive competitors. Where competition among males for mates is intense, high resource holding potential (RHP) individuals may control access to reproducing females^1^ for example by mate guarding them during their fertile period^2–4^. However, this control is often limited because females may attempt to escape their mate guard to mate with rival males, or because competing males adopt sneaky alternative reproductive tactics^5,6^. One way that males may seek to gain an advantage is by ensuring they are the first to copulate; an early mating advantage has been observed in a taxonomically wide range of animals^7–14^. However, early mating may not necessarily translate into a fertilisation advantage. Sperm storage mechanisms extend the window for sperm competition to take place, eroding the fertilisation advantage sperm may have when given a head start^15–17^. Although males can invest in competitive sperm phenotypes to achieve fertilisation ahead of rivals, they may face an uneven post-copulatory playing field on which to compete. Females can bias fertilisations to the stored sperm of preferred males via cryptic female choice mechanisms^18,19^, further reducing male reproductive control. Sperm storage and cryptic female choice may explain why many studies have failed to detect any significant effect of copulation order on fertilisation success across a taxonomically wide range of animals^20–25^.

Indeed, later copulating males can have an advantage in achieving fertilisation^26–31^. If cues of previous copulations are recognisable, later maters may make flexible competitive adjustments in copulatory behaviour^32,33^ and investment in ejaculates^34^. Later copulators may also benefit from morphological adaptations designed to remove rivals’ sperm^35,36^. Therefore, even if high RHP males largely dominate access to females, any rival copulations may dramatically reduce their siring success. To translate advantages in access to females into reproductive success, males may need to engineer an early mating advantage themselves, for example by guarding females to prevent rival copulations^24,37^, or through the use of copulatory plugs^38^.

Most pre-existing studies of an early mating advantage have focussed on captive animals^7–12^. These captive studies have provided powerful insights, specifically allowing experimental manipulations of mating order that are extremely challenging in the wild. However, any mating advantage evidenced in captivity occurs under relatively benign conditions in which individuals may not face the same trade-offs between reproductive investment and future survival and fecundity faced in wild populations^39,40^. For example, investment in sperm competition or cryptic mate choice may be different in wild populations in which individuals are at risk of predation, starvation, disease, and other forms of extrinsic mortality. More studies of wild populations would be useful to assess the prevalence and adaptive value of early mater advantages in the wild, yet these are relatively scarce due to observational challenges, such as detecting copulations that are often infrequent and can occur out of sight. Moreover, linking copulations to paternity requires a marked population for which there is a genetic pedigree (e.g. different wild ground squirrels^14,41–43^, ring-tailed lemurs *Lemur catta*^13^)*.*

Here, we address this knowledge gap by investigating an early mating advantage in wild banded mongooses. Banded mongooses live in mixed sex groups with a core of 2–5 female and 4–12 male breeders^44^ that breed 2–4 times per year^45,46^. Older males guard access to promiscuous females over the course of synchronised group oestrus events that last less than a week^45^.

Despite mate guards, rivals can adopt alternative sneaky ‘pestering’ tactics^47,48^ to gain copulations when guarders are distracted while avoiding direct fights^3^. Females have also been observed attempting to escape their guards^45^, with pestering males waiting to take advantage of such opportunities^3,49^. Although these pesterers do gain some copulations, 83.7% of observed copulations are attempted by mate-guarding males^45^. Guarding males can only secure one female at a time, so their paternity share of the group litter can be seriously impacted if rival males have copulation success. An early mating advantage would suggest post-copulatory competition is reduced, providing some insurance for when pestering males successfully sneak copulations. Secondly, we make the first ever assessments of the functional properties of banded mongoose ejaculates and evaluate if these properties indicate the presence of a copulatory plug. Copulatory plugs could allow males to counteract females’ promiscuity if they cannot prevent subsequent copulations by rivals. Indeed, female promiscuity is a key driver in the taxonomic presence of copulatory plugs. Copulatory plugs are largely absent in monogamous mating systems or where there are mechanisms to lock males and females in extended copulations^38,50^. Copulatory plugs may provide the proximate mechanisms underlying any evidence of an early mater advantage found in banded mongooses.

To assess an early mating advantage we use 17 years of paternity data and behavioural observations from a wild population of banded mongooses in Uganda^49^. Since copulations are rarely observed in our wild study system, we test for an early *interaction* advantage using daily behavioural observations. We predicted that earlier interactions with females during group oestrus events would translate into greater siring success. As such, we provide a rare test of an early mating advantage in free-living animals. An early interaction advantage would be effected by the presence of copulatory plugs, which would be supported by ejaculates with viscous and sticky properties as previously described in other systems^51–55^.

## Methods

### Study population

Data were collected using a wild banded mongoose population living on the Mweya Peninsula, Queen Elizabeth National Park, Uganda. Comprehensive life-history data has been collected on this population since 1995, including births and deaths. Data from 7 groups were used in this study. A genetic pedigree based on 43 microsatellite loci has been collected from 2003 to 2020 (see references for how the pedigree is obtained^56,57^. In brief, DNA was extracted from 2mm tissue samples taken from tail tips. These samples were genotyped using multiplex PCRs (Qiagen™ Multiplex PCR Kit, UK) at up to 43 microsatellite loci. Parentage analysis used likelihood based methods conducted using MasterBayes^58^ and COLONY^59^.

Banded mongoose groups are highly male-skewed due to female-biased mortality^49^. Compared to females that typically start reproducing at one year of age, male skew delays reproductive onset in males due to waiting in a ‘queue’ for reproductive positions^3^. This delay means group demographics typically include a number of reproducing and non-reproducing adult males. Females have on average 1.92 pups that survive to be genotyped per litter of which 70.5% (225/319) are sired by a single male.

### Historic observation of reproductive behaviour

Since 2003, reproductive activity during group oestrus events has been closely observed. Groups were visited daily when oestrus events were expected. When signs of oestrus in the group appear, such as the first observations of mate guarding behaviour by males, during daily group visits 20 min focal behaviour observations are carried out on each reproducing female in the group. These 20 min focals accurately capture the reproductive behaviour of males for that day of oestrus. The consistency between focal observations and behaviour outside of these sampling periods has been informed by following these groups for 5 h a day for the last 17 years (> 100,000 h of observations in total). The mean number of data collection days per oestrus event was 3.8. The identities of males engaging in reproductive behaviour towards each female were noted. Guards were defined as the single male that closely followed the female over the focal duration. Additional males may follow the guarded pair and attempt to opportunistically sneak copulations; these were defined as pestering males. Females can have multiple pesterers at a time. Copulations themselves are rarely observed during these visits. Daily visits and focal observations continued until the last female ceased oestrus. Oestrus events were defined as the period between the first female going into oestrus until oestrus in all females in the group ceased. Whether a male guarded or pestered over the course of an oestrus event was defined according to their average behaviour towards the female (if guarded ≥ 50% of days defined as guard, if pestered > 50% of days then defined as a pesterer).

The banded mongoose gestation period is around 9 weeks (pooled data from^45,46^). There were 78 cases where a litter could be linked to an oestrus event where the mother was observed interacting with at least 2 unique reproducing males including the sire, verified with pedigree data.

### Statistical analysis: testing for a historic early mating advantage

For each dyadic interaction between reproducing males and females associated with the 78 litters a binomial success (or fail) was determined if the male succeeded in siring the female’s offspring, modelled with binomial error distribution using Bayesian inference with JAGS MCMC^60^ in R. We used uninformative priors. Copulation order could not be used to test for an early mating advantage as copulations themselves are rarely observed. Potential copulation order was inferred from the day males were first seen to guard or pester a given female, which was fitted to the model. To control for competitive pressure, the number of rival pesterers or guarders that interacted with the same female was also fitted. To control for the success of the two reproductive strategies (guarders versus pesterers), the strategy adopted by a male towards a given female was fitted as a binomial variable. To assess if the effect of order was influenced by reproductive state or number of competitors (guarders or pesterers), interaction terms were initially included, but did not have a credible effect and were dropped from the final model. This analysis included 108 individual males (80 successful sires) who interacted with 47 individual females during 48 oestrus events, included as random intercepts in the model to account for repeated measures.

### Ejaculate collection

Males were caught using bated Tomahawk traps (Tomahawk live Trap Co., Tomahawk, Wisconsin, USA). Isoflurane (5%) (IsoFlo, Abbott Laboratories) was used to anesthetise males, reduced to 2% once under anaesthesia. Ejaculates were collected using electro-ejaculation^61^. The male was placed on its back and the penis was cleaned gently with wetted cotton wall. Lubricant (KY jelly) was inserted into the rectum with a pipette. A probe covered in the same lubricant was inserted 1.5cm into the rectum. Two electrodes at the end of the probe faced upwards to stimulate the prostate. A series of 5 electrical stimulations were transmitted through the prope using an audio amplifyer (QTX KAD-2BT). Each series comprised 17 half-second bursts with half-second breaks inbetween that progressively increased in intensity (0.5–5mA; see Supplementary Information for full song details), controlled through audacity. A multimeter (Kewtech KT117) was monitored throughout to ensure current remained within the expected range. Ejaculates were handled with wooden cocktail sticks. Ten ejaculates from different males were collected for viscosity tests.

### Ejaculate viscosity tests

We used standardised viscosity tests carried out in previous research^62^. Ejaculates were aspired into a wide bore 5ml pipette. The pipette was held to allow the ejaculate to separate with gravity. Viscosity was measured as the length of each thread that separates from the rest of the ejaculate.

### Ethics & transparency

Prior approval of all work was received from Uganda Wildlife Authority (UWA) and Uganda National Council for Science and Technology (UNCST). All procedures adhered to the ASAB Guidelines for the Treatment of Animals in Behavioural Research and Teaching and were approved by the Ethical Review Committee and Animal Welfare Review Board of the University of Exeter (eCORN000006). This study is reported in accordance with ARRIVE guidelines 2.0

## Results

### Early interaction advantage

**Fig. 1:**
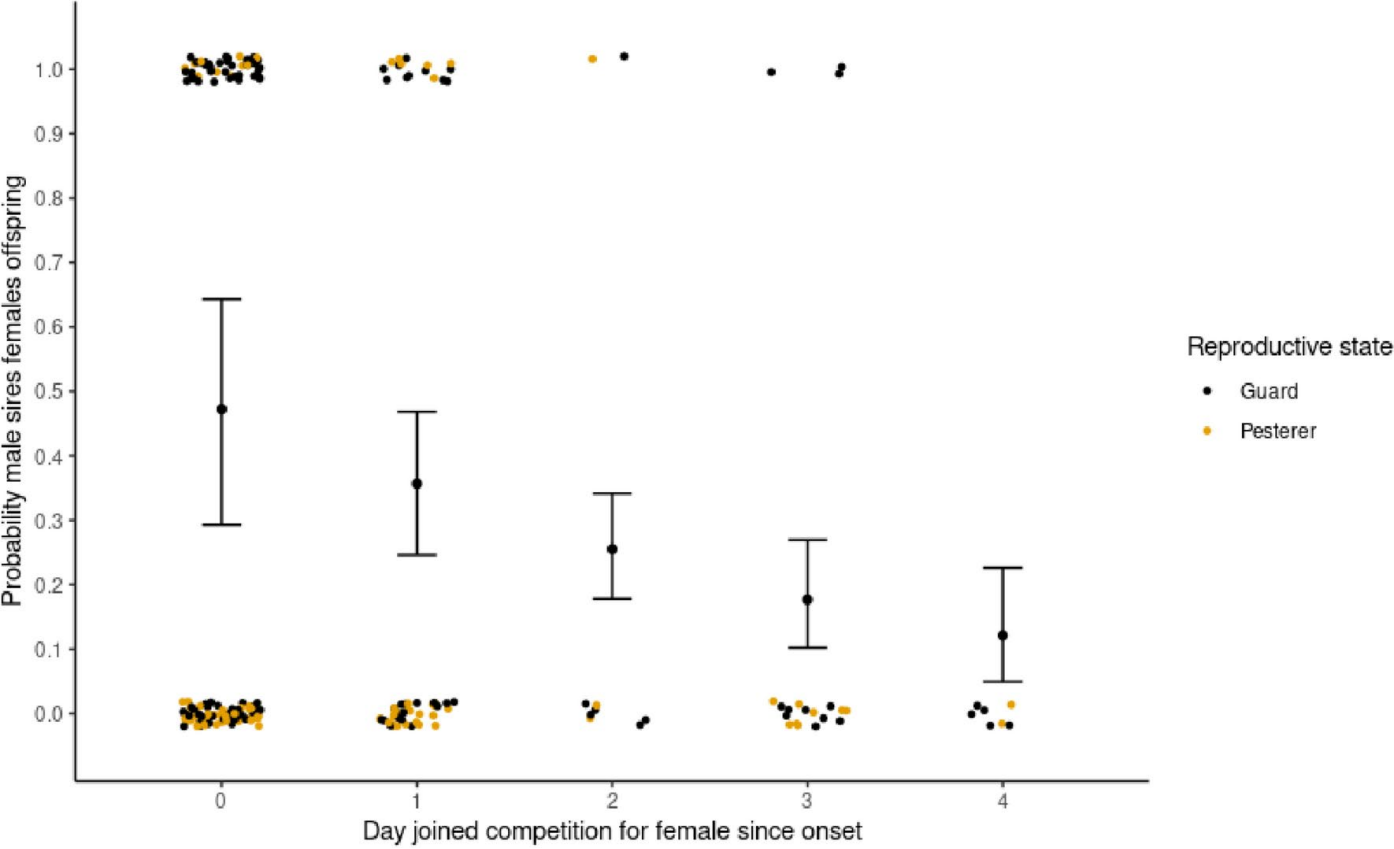
The effect of day a male started pursuing a female since onset of oestrus on the male’s probability of siring the female’s offspring. Error bars represent the mean, lci and hci of the predicted posterior distribution of the model. Points represent raw successes and failures at siring for each case where a male pursued a female; colours represent the reproductive state of the male (guard in black, pesterer in orange).

While accounting for the effects of reproductive competition (number of separate pesterers and guarders that pursued the same female), and males’ own reproductive behsaviour (guarder or pesterer), males that interacted with a given female earlier in the oestrus event had a significant siring advantage (Table 1). From an approximate 50% of siring a female’s offspring (Fig. 1, mean = 0.468, lci = 0.337, hci = 617), each day of delay decreased the chance a male would sire the female’s offspring (Fig. 1), independent of competition for the female and the male’s own reproductive behaviour. There was no evidence of an interaction between the reproductive tactic of the male and when they started interacting with a female, suggesting the above early interaction advantage was present for guarders and pesterers.

**Table 1:** Model outputs for the probability a male sires a female’s offspring.

Term	Effect	sd	2.50%	50%	97.50%	Rhat	f	Overlap0
Intercept	− 2.05	0.44	− 2.94	− 2.02	− 1.27	1	1	–
Pestering competitors	− 0.11	0.22	− 0.51	− 0.12	0.35	1	0.70	Yes
Guarding competitors	− 0.49	0.23	− 0.96	− 0.49	− 0.06	1	0.99	**–**
Tactic of male	1.19	0.49	0.27	1.18	2.25	1	0.99	** + **
Day joined competition	− 0.46	0.25	− 0.96	− 0.46	− 0.01	1	0.98	**–**
Random effect	Male.id	Female.id	Oestrus.code					
sd	0.24	0.012	0.012

Means (effect), Credible intervals (0.025,0.975), and median (50%) effects for each covariate are sampled from the untransformed posterior distribution of each model. Effect sizes are on the logit scale. f is the proportion of the posterior distribution with the same sign as the mean. Overlap 0 shows whether 0 overlaps with the range of 2.5% and 97.5% quantiles of the posterior distribution for each fitted parameter, with bold covariates those that had a significant effect (no overlap). Where there was no overlap, the direction of the effect is given under ‘Overlap0’. Rhat is a measure of chain convergence (< 1.1) (Gelman & Rubin, 1992). The standard deviation of the three random effects, Male ID, Female ID, and Oestrus event ID, is given for each model.

### Ejaculate sample description and viscosity

**Fig. 2:**
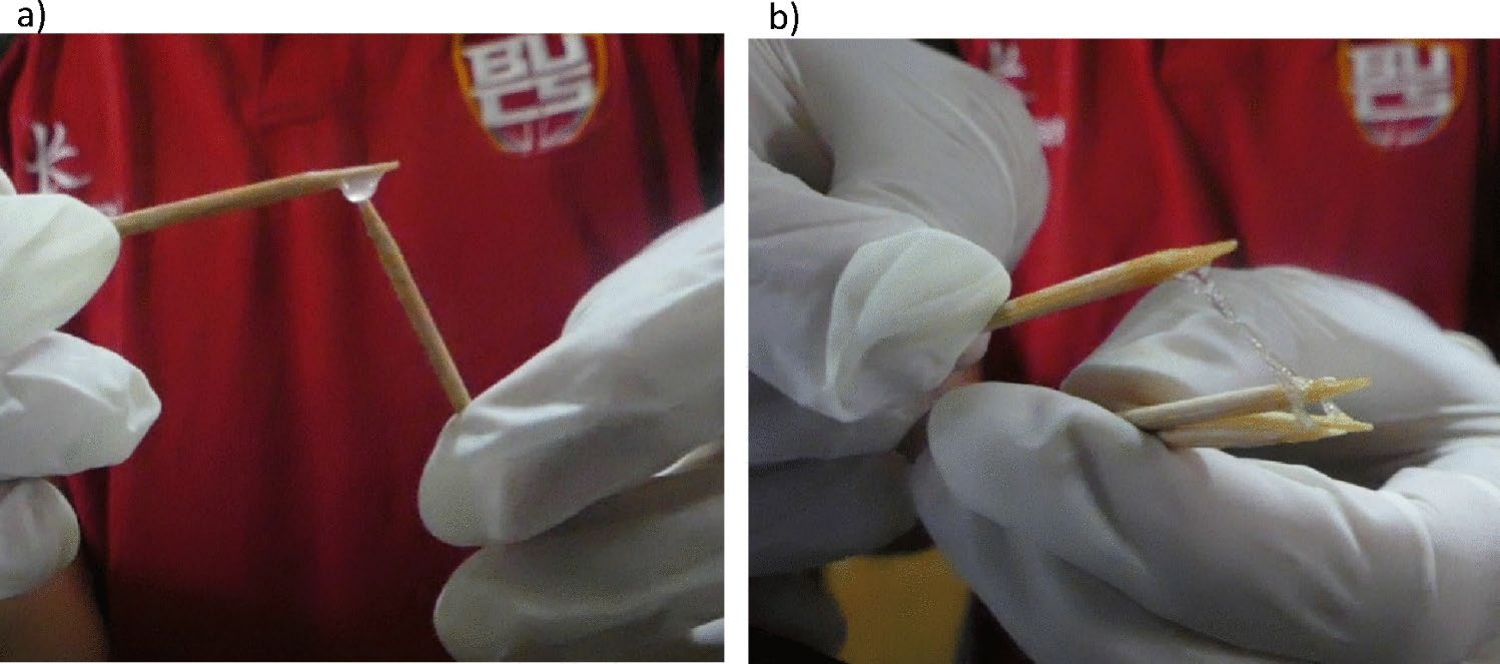
Images of banded mongoose ejaculates. Cocktail sticks were used to handle the ejaculates. (**a**) Ejaculates form a globule and have remarkable elastic, glue-like properties; and (**b**) continue to adhere to the cocktail sticks when stretched.

Banded mongoose ejaculates were invariably highly viscous and not solid. Ejaculates did not lose this viscosity over time, failing to liquify. These ejaculates remained too viscous for standard viscosity tests, samples aspired into a wide bore 5ml pipette (n = 10) failed to separate due to gravity. The highly viscous ejaculate forms a sticky globule with remarkable elastic, glue-like properties (Fig. 2).

### External signs of copulatory plugs

Over the last 30 years of observation in the field, external signs of copulatory plugs have not been seen. Females are rarely captured during oestrus events to avoid disruption of reproductive behaviour. Where these captures have been necessary during historic experiments, and more recently for the purpose of this study, obvious signs of external copulatory plugs have not been seen.

## Discussion

Our study provides evidence of an early interaction advantage for male banded mongooses siring offspring. There was no evidence this early interaction advantage was specific to guards, suggesting guards and pesterers both benefit. Although observed copulations could not be used, our unique long-term dataset provides indirect evidence of an early mating advantage. The reliance on indirect evidence was necessary due to the rarity with which copulations are observed in the wild, often occurring briefly and out of sight. Direct observation of copulations is a challenge common to other wild study systems^14^. Assessments of an early or late mating advantage come almost exclusively from captive study systems, such as in birds^9,30,63^, fish^10^, mammals^8,21,24,26,29,31^, reptiles^20^, and invertebrates^7,11,12,22,25,28,64^. In this study we present indirect evidence of an early mating advantage in a free-living study system, to our knowledge shown before only in different ground squirrel species^14,41−43^. Ejaculates also had properties consistent with previous descriptions of copulatory plugs in other species^51–55^, being highly viscous, sticky, and failing to liquify even after extensive periods at room temperature. The viscosity of all ejaculates was too high for standard viscosity tests used for human ejaculates^62^. Although there were no external signs of copulatory plugs, we tentatively suggest that the sticky ejaculate may internally adhere to the female reproductive tract in such a way to reduce the success of subsequent copulations from rivals. By mating earlier, guards and pesterers may give their sperm a significant head start in fertilising a female’s^7–14^ insured by the use of copulatory plugs.

Independent of the presence of copulatory plugs, mating early may mean enough time passes between copulations for fertilisation to have already occurred^7–14^. Mate-guarding after securing an early copulation may delay or prevent post-copulatory sperm competition by ensuring a substantial time gap between a guard’s own copulation and subsequent rival copulations. Similarly, pesterers who have successfully sneaked a copulation may benefit from continuing to harass a guarded pair if it delays copulations by the mate guard, disrupting subsequent post-copulatory competition. However, female banded mongooses are often followed constantly by males during oestrus, so the time between copulations with rival males may not always be sufficient to ensure an early mating advantage. Additionally, any sperm storage^15–17^ or cryptic female choice mechanisms^18,19^ that may be present could neutralise a headstart, which may explain why many other studies have found no advantage for earlier mating in other systems^20–25^.

Copulatory plugs may present the proximate mechanism by which males achieve an early mating advantage. Copulatory plugs are effective at counteracting female promiscuity, for example leading to an observed early male mating advantage in bank voles (*Myodes glareolus*)^65^ and ring-tailed lemurs (*Lemur catta)*^13^. Experimental tests have further shown the effectiveness of copulatory plugs at securing paternity. The first mating advantage of laboratory mice was nullified when an enzyme critical in the coagulation reaction of their copulatory plugs, Tgm4, was knocked out^66^. Similar results were found when mating plugs were physically removed from laboratory mice, secondary maters becoming much more successful^67^. Copulatory plugs could act as an insurance policy for male banded mongooses if they cannot prevent subsequent copulations by rivals, engineering an early mating advantage as indicated by our results.

Where copulations have been recorded they have largely been linked to males who were observed to be reproductively active during focals, for example past research has identified that guards account for 83.7% of observed copulations or mounting attempts in banded mongoose groups^45^. Therefore, we are confident that focal observations of reproductive behaviour of males as guards, or pesterers, is a good indication of copulations. However, our evidence for an early mating advantage, based on the earliest observed interaction, has some limitations. Additional factors may explain the fertilisation success of early interactors not explained by early copulations or use of plugs. One factor is female choice. Females should be incentivised to choose high quality mates as sons may inherit fathers’ competitiveness, increasing the genes that mothers will pass on to the next generation^68–70^. Males that successfully guard females early in the oestrus event may signal their quality to these females, and have their copulation attempts more often accepted or sperm favoured by cryptic choice mechanisms. Additionally, by interacting earlier these males have the opportunity to copulate more frequently over the oestrus event. More time interacting with the female may also allow these males to select more fertile windows to copulate, such as during or a couple days prior to observation^71^. However, ovulation may be induced by copulations in banded mongooses as seen in other *herpestidae*^72^, and copulation or mounting frequency has been previously shown not to vary significantly over the course of oestrus events^45^, suggesting together that selective copulations by males to target more fertile windows may not be applicable. Additionally, female choice for competitive males, or higher frequency of copulations, which would be associated with earlier interactions may only be applicable to explain an early mating advantage for guards. Pesterers had a similar early interaction advantage, but such males lack the competitiveness of guarding males for female choice to act upon, and opportunistic sneaky copulations may not allow these males to copulate frequently or pick fertile windows. The early interaction advantage we have found points to the likelihood of an early mating advantage, perhaps facilitated by use of copulatory plugs. However, we cannot rule out a role for female choice and/or copulation frequency.

An early mating advantage would have implications for male mate choice. For male mate choice to evolve males should be forced into time-limited simultaneous assessments of females^73,74^, likely the case in banded mongoose with synchronised short oestrus events^3,49^. Female banded mongooses vary in their fecundity; larger, older females produce more offspring and these females are targeted by the oldest males^3^. Females also vary in their relatedness to males, and guards avoid inbreeding through their mate choices^56^. Any errors in initial mate choice may mean males lose fitness by missing siring opportunities with the most fecund or most genetically compatible females. Indeed males may be discouraged by the cues of rival copulations; a taxonomically wide range of studies shows that males reduce their probability of copulating with females who have recently copulated with rivals^75–81^, including specific discrimination against females who have a copulatory plug in place^81–83^. Such evidence comes largely from studies of invertebrates^80,84^. Whether males in free-living vertebrates widely discriminate against females recently mated by rivals requires future research. Overall, an early mating advantage would heighten the importance of initial mate choices by males in banded mongoose groups.

## Conclusion

Using long-term behavioural data on a wild population of banded mongooses, we provide indirect evidence of an early mating advantage in a free-living wild system. By mating early, males may establish a significant head start in sperm competition over rivals. This advantage is likely secured by mate guarding behaviour to block or disrupt rival copulations. We also speculate that males engineer an early mating advantage by using copulatory plugs as an insurance policy against the risk that rivals subsequently obtain copulations. An early mating advantage may intensify initial male-male competition, therefore heightening selection for male mate choice to secure access to the most fecund or genetically compatible females.

## Supplementary Information

Supplementary Information 1.

## Data Availability

Data is available online https://github.com/GrahamBirch/Early-mating-advantage.

## Code availability

Code is available online https://github.com/GrahamBirch/Early-mating-advantage
